# Validation of UK Biobank data for mental health outcomes: A pilot study using secondary care electronic health records

**DOI:** 10.1016/j.ijmedinf.2022.104704

**Published:** 2022-04

**Authors:** Zhenpeng Li, Andrey Kormilitzin, Marco Fernandes, Nemanja Vaci, Qiang Liu, Danielle Newby, Sarah Goodday, Tanya Smith, Alejo J Nevado-Holgado, Laura Winchester

**Affiliations:** a Department of Psychiatry, University of Oxford, Oxford OX3 7JX, UK; b 4youandme, Seattle, WA 98121-1031, USA; c NIHR Biomedical Research Centre, Oxford Health NHS Foundation Trust, Warneford Hospital, Warneford Lane, Oxford OX3 7JX, UK

**Keywords:** Mental health, UK Biobank, Linkage studies, Validation study, Data resource, Neuro-epidemiology, UKB, UK Biobank, CRIS, Clinical Record Interactive Research, EHR, Electronic Health Record, PPV, Positive Predictive Value, ICD-10, International Classification of Disease 10th Revision

## Abstract

UK Biobank (UKB) is widely employed to investigate mental health disorders and related exposures; however, its applicability and relevance in a clinical setting and the assumptions required have not been sufficiently and systematically investigated. Here, we present the first validation study using secondary care mental health data with linkage to UKB from Oxford - Clinical Record Interactive Search (CRIS) focusing on comparison of demographic information, diagnostic outcome, medication record and cognitive test results, with missing data and the implied bias from both resources depicted.

We applied a natural language processing model to extract information embedded in unstructured text from clinical notes and attachments. Using a contingency table we compared the demographic information recorded in UKB and CRIS. We calculated the positive predictive value (PPV, proportion of true positives cases detected) for mental health diagnosis and relevant medication.

Amongst the cohort of 854 subjects, PPVs for any mental health diagnosis for dementia, depression, bipolar disorder and schizophrenia were 41.6%, and were 59.5%, 12.5%, 50.0% and 52.6%, respectively. Self-reported medication records in UKB had general PPV of 47.0%, with the prevalence of frequently prescribed medicines to each typical mental health disorder considerably different from the information provided by CRIS.

UKB is highly multimodal, but with limited follow-up records, whereas CRIS offers a longitudinal high-resolution clinical picture with more than ten years of observations. The linkage of both datasets will reduce the self-report bias and synergistically augment diverse modalities into a unified resource to facilitate more robust research in mental health.

## Highlights

• Linking UK Clinical Record Interactive Search (UK-CRIS) and UK Biobank.
• Comparison of diagnosis for 854 individuals present in both datasets.
• Electronic health records from UK-CRIS used to understand self-reported data in UK Biobank.

## Graphical abstract

**Figure:**
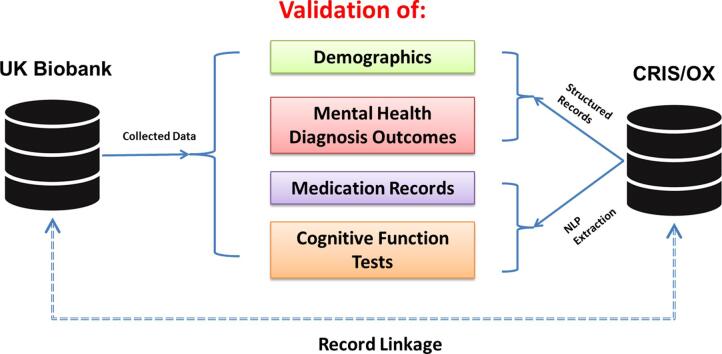

## 1 Introduction

Almost 14% of the global burden of diseases is related to neuropsychiatric disorders including common mental health disorders such as depression [1]. These disorders are measured in different cohorts such as UK Biobank (UKB). UKB is a large population-based data resource (>500,000 participants) with a wide variety of exposures involving demographic, lifestyle, environmental and health information for the assessment of determinants of various life-threatening and disabling conditions, including mental health disorders. The UKB includes baseline data on a series of cognitive testing and physical measures, along with follow-up data of diagnostic disease outcomes, repeat cognitive testing, self-reported questionnaires, genotyping and multimodal imaging to incorporate comprehensive information and facilitate longitudinal study [2], [3]. Whilst UKB could be a great resource for mental health research, the reliability and validity of the included measures in comparison to physician confirmed information remains largely unknown. Validation studies to date focused only on one aspect of the data accuracy, such as the precision of diagnosis for a certain disease [4], [5]; many used adjudicated outcomes by clinicians as their gold standard which is labour intensive [5]; and very few studies were able to assess the reliability of recorded medication or results of cognitive tests in the UKB.

The Clinical Record Interactive Research (CRIS) platform is a large secondary care network based in the UK incorporating over 2.7 million de-identified patient records designed to facilitate advanced research into mental health [6]. CRIS Oxford (CRIS/OX) is one of the twelve NHS mental health trusts within the network providing an avenue to perform analysis on the electronic health records (EHR) collected from Oxford Health NHS Foundation Trust (OHFT) [7]. CRIS/OX unlocks and transforms the EHR stored in trust systems to provide a pseudonymised resource allowing researchers and clinicians to investigate hypotheses and identify patient cohorts. It provides a unique opportunity to validate the quality of the relevant data recorded in the UKB.

In this paper, we present the first integrated study of data from the UKB for mental health with the CRIS research platform. We describe the results of the pilot validation study with the aims of comparing the individual patient data in demographics, diagnoses, medication records, and relevant cognitive tests using the CRIS as the reference standard. The objective of this comparison was to understand on the quality of data, the breadth of missing data and associated biases, and provide advice on employing UKB data for conducting research on mental health.

## 2 Methods

### 2.1 Cohort determination

**Fig. 1:**
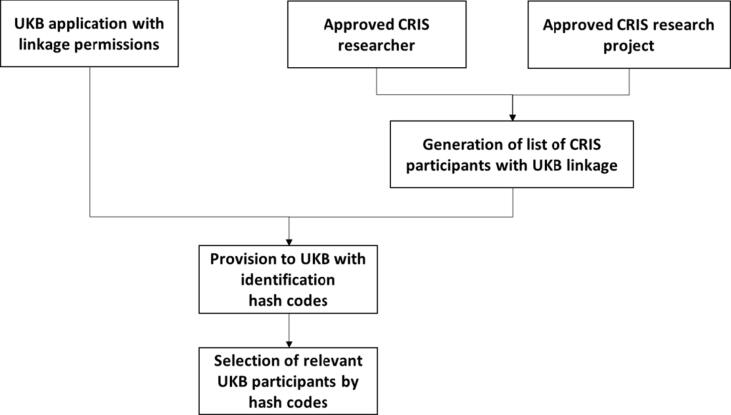
Flow Chart for the Procedure of record linkage between UKB and CRIS/OX.

CRIS has established a clinical data linkage service (CDLS), which provides assistance for researchers to link patient records from diverse sources at the individual level, whilst ensuring the confidentiality of patient information complied with legal and ethical rights. The procedure for record linkage between the UKB and CRIS is shown in Fig. 1 (details in Appendix A).

### 2.2 Data source and extraction

The objective of the study is to compare variables which are either co-existing from both data sources or should theoretically be complementary. For UKB, we employed a 2019 data release (UKB25084). Briefly, the demographic information of participants together with the self-reported diagnosis and prescribed medications was collected during their baseline assessment between 2006 and 2010 and updated in follow-up visits from 2012 to 2018. Hospital inpatient data included retrospective records from 1997 with annual update until 2018. Multiple resources were incorporated to produce diagnosis outcomes in 2017 (Appendix B).

CRIS/OX enables users to access rich information recorded by mental health professionals in secondary care settings. Both structured and unstructured data are collected. Patient demographics and diagnostic information are recorded in structured format. A range of information is embedded in free text clinical notes and attachments dated between 2000 and 2019. Clinical notes contain ward round notes, phone calls and clinical observations. Attachments include letters from general practices (GPs), test results, referral and clinic letters or reports. These files are of significant value to medical research as they contain rich information about patients. In the current study, the records of prescribed medications along with the cognitive test results including Health of the Nation Outcomes Scales (HoNOS), Montreal Cognitive Assessment (MoCA) scores and Mini-Mental State Examination (MMSE) scores were extracted by means of clinical natural language processing (NLP) models [8], [9], [10], [11], [12]. We used an NLP text extraction system designed for CRIS/OX and achieved an F1 score of 92.8% and 98.03% for diagnosis and medication respectively [13].

### 2.3 Statistical analysis

We compared four main aspects of the UKB data against the clinical data in CRIS/OX including demographic data, diagnostic outcomes, medication records and results of cognitive tests. For statistical comparison, the positive predictive value (PPV) [5] was employed to evaluate the quality of the information from the UKB. The PPV corresponds to the ratio of true positives over the combination of true and false positives (for demographic information, diagnosis outcomes and medication records), with confidence intervals determined by Clopper–Pearson (exact) method [14]. Besides, each type of information has its intrinsic characteristics, which requires a distinct analytic method.

For demographic data, two elements: gender and ethnic groups were examined with contingency tables whereby each row represented the information from the CRIS/OX as an observed class whilst each column represented the information from the UKB as a predicted class. The corresponding PPV for each category were calculated. For date of birth, given that CRIS/OX systematically truncated all date of birth to the first day of the month for confidentiality reasons, only the year and month of birth were extracted for validation. For ethnic groups, to harmonise the different encoding system used in UKB and CRIS/OX, a universal list with five broad ethnic groups was adopted (as recommended for use by the UK government) [15].

For diagnosis outcomes, we examined any mental health disorder and in particular focused on four specific mental health disorders (dementia, depression, bipolar disorder and schizophrenia). Firstly, we assessed the accuracy of the self-reported data by comparing against the relevant information retrieved from multiple sources within UKB. Then we took a further step to evaluate the PPV of mental health diagnoses recorded in the UKB. The true positives for PPV evaluation contain two groups of individuals: (1) individuals with no records of mental health disorders on either side; (2) individuals with at least one record of the specific mental health disorder also identified from CRIS/OX. False positives were subjects with presented mental health disorders in UKB but not indicated from CRIS/OX. Diagnostic disease outcomes in UKB are acquired from multiple sources: Self-report diseases, Hospital Episode Statistics (HES) inpatient data, Mortality data and Algorithmic-defined outcomes where available. The International Classification of Disease 10th Revision (ICD-10) coding was used as a common comparison (Appendix C). Briefly, ICD-10 codes are in the format of a single alphabetic character followed by 3 digits. Such format enables the creation of a hierarchical structure for diagnostic data; i.e., splitting from top level which represents the general diagnosis to bottom level which denotes a very specific diagnostic case; to demonstrate the different levels of precision for diagnosis. When considering matching records of high diagnostic precision without loss of generality, diagnosis data in ICD-10 at medium level (medium level denoted by 3-character category: 1 alphabet with 2 digits) from both sides was adopted for comparison.

For medication records, true positives also involve two scenarios: (1) individuals having no records of medications related to mental health disorders on either side; (2) individuals with at least one drug record on mental health provided by UKB also detected from CRIS/OX. False positives were those subjects without testimony in CRIS/OX to verify the records of medications for mental health disorders in UKB. UKB participants were asked about their regular medications at baseline assessment (2006–2010) and follow-up visits, with the reported medication names recorded by nurse interviewers and transformed into digital codes according to a pre-defined code list. On CRIS/OX, the prescribed medications were embedded in the full-text electronic medical records, including clinical notes, hospital and outpatient correspondence as well as investigation results. Natural language processing (NLP) was performed on text files to extract medication [13]. A preliminary step to harmonise medication records based on chemical names was performed for both UKB and CRIS/OX prior to execution of the validation process.

## 3 Results

In the study, a cohort of 854 subjects was identified with records in both UKB and CRIS/OX. In this cohort, 492 (57.6%) were female, the median birth year was 1947 (range 1937–1969), the median age of recruitment was 58 years (range 41–70) and 45 (5.3%) participants died during follow-up.

### 3.1 Comparison of demographic data shows an accurate linkage between UKB and CRIS/OX

**Fig. 2:**
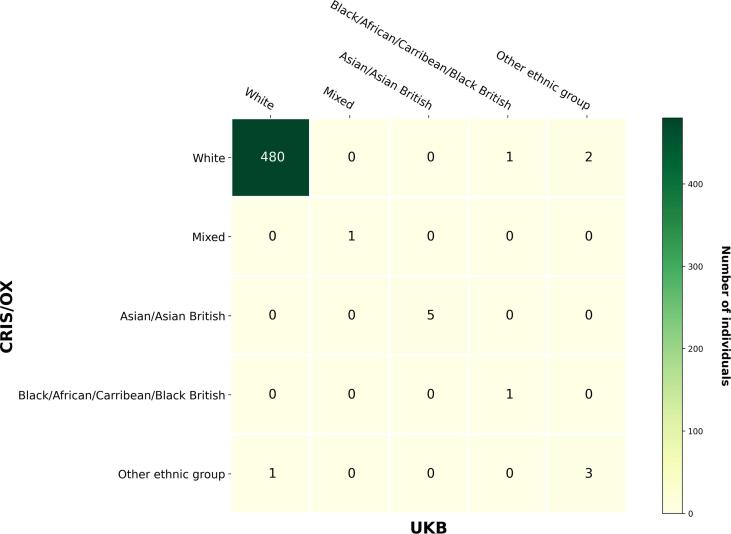
Consistency of Ethnicity Demographics between UKB and CRIS/OX.

Firstly, we compared the two datasets to understand whether the matching process was successful. The Demographic information, including gender (Appendix D), the year and month of birth from both data sources were perfectly matched with no discrepancy, resulting in a PPV of 100%. Fig. 2 illustrates the contingency table of the matched results on ethnic groups between UKB and CRIS/OX. Of all 854 individuals, 355 had no ethnicity records in CRIS/OX. Amongst which, 336 (94.7%) were recorded of “White” ethnic group in the UKB. Of the 494 individuals with ethnicity data available on both sides, 490 (99.2%) were documented with matched information. The majority of the matched records were categorised into the generic ethnic group of “White”.

### 3.2 Consistency of diagnosis outcomes

#### 3.2.1 Understanding of self-reporting diagnosis compared to any diagnosis outcome variables in UKB

Overall, self-reporting accounted for less than half of the final diagnosis using multiple resources for each of the specific mental health disorders in the UKB. Depression has a higher proportion of self-reported cases (48%) whereas dementia (1.4%) has the lowest (Table 1). Self-reporting variables were compared to any diagnosis counterparts to understand whether they were confirmed. A considerable proportion of individuals with self-reporting diagnosis of mental health disorders were not identified using the other diagnosis variables (Table 2), especially for depression (44.1%).

**Table 1:** Self-reported Cases Compared to Any UKB Diagnosis Measures.

	Depression	Dementia	Bipolar Disorder	Schizophrenia
Self-reported Cases in UKB	200	3	44	13
Total Cases by Any Diagnosis Measures in UKB	417	217	116	39
Proportion of Self-reported cases	48.0%	1.4%	37.9%	33.3%

**Table 2:** Total Participants in UKB where Self-reporting is the Single Measure to Define Diagnosis.

	Depression	Dementia	Bipolar Disorder	Schizophrenia
Participants with Only Self-reported Cases in UKB	123	0	17	5
Participants with Diagnosis from Any Diagnosis Measure in UKB	279	79	62	19
Proportion of Participants with Self-reported only diagnosis	44.1%	0.0%	27.4%	26.3%

#### 3.2.2 Consistency of diagnosis outcomes in UKB by comparison to CRIS/OX

**Fig. 3:**
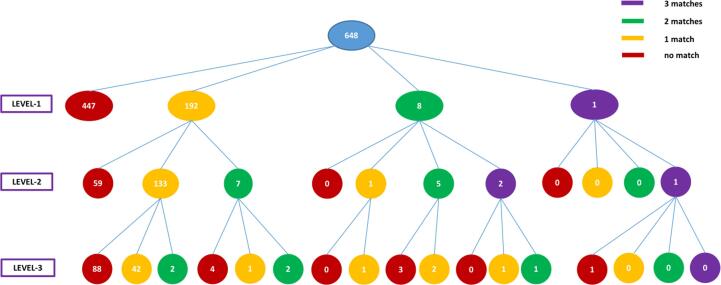
Hierarchical Structure of Matched Diagnostic Data from UKB and CRIS/OX at Different ICD10 Levels: Comparison of ICD10 Diagnostic Data demonstrating the contrast in overlap at different description levels, coloured by number of matches indicating whether match is supported by multiple record entries for a single participant.

Of all 854 individuals, when the resources were compared, the number of matched records for each individual at different levels of diagnostic precision was examined. This was calculated from 648 individuals with records of any mental health disorders in at least one of the data sources (Fig. 3), with the level of precision increasing from LEVEL-1 to LEVEL-3 (Appendix C).

Briefly, 201 (23.5%) individuals had matched diagnosis data of any mental health disorders at the top level (LEVEL-1), followed by 149 (17.4%) matched individuals and 52 (6.1%) matched individuals at medium level (LEVEL-2) and bottom level (LEVEL-3), respectively. Moreover, at each level of diagnostic precision, even in those with matched records on the presence of any mental health disorders (LEVEL-1), most individuals had one diagnosis matched in the two data sources.

The remaining 206 (24.1%) individuals had no records relevant to mental health disorders in either UKB or CRIS/OX, indicating that although they are likely to have a mental health issue which required secondary mental health care, they do not have a clear diagnosis in either data source.

**Fig. 4:**
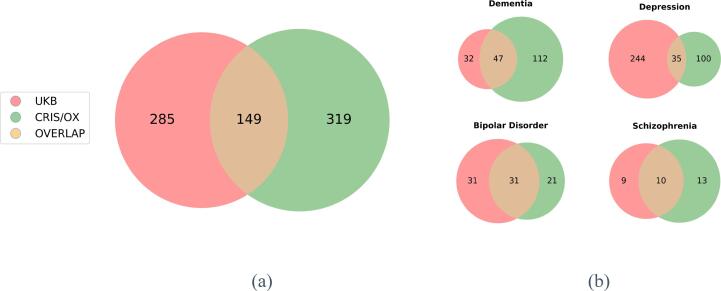
Agreement of Diagnostic Outcomes from UKB and CRIS/OX: (a) Count of overlapping participant diagnosis between UKB and CRIS/OX when comparing data resources for all diagnostic outcomes; (b) Count of overlapping participant diagnosis for four specific mental health disorders.

The overlap of individuals with confirmed diagnostic cases are of particular interest. Fig. 4a presents an overview of agreement on any mental health disorders between the UKB and CRIS/OX at LEVEL-2. Together with the 206 subjects with agreement on having no diagnostic records of mental health disorders, 355 (41.6%, 95% CI: 38.2% − 45.0%) had matched diagnosis with a PPV of 34.3 (95% CI: 29.8 - 39.0%) for UKB.

Of the four main mental health disorders of interest, the diagnosis of depression in the UKB had the lowest PPV (12.5%, 95% CI: 8.9–17.0%, Fig. 4b), followed by bipolar disorder (50.0%, 95% CI: 37.0–63.0%), schizophrenia, (52.6%, 95% CI: 28.9–75.6%) and dementia (59.5%, 95% CI: 47.9––70.4%, Fig. 4b).

Of note, in addition to sub-optimal PPVs, in absolute terms, the UKB also tended to have more individuals with apparent diagnosis of depression and much fewer with the diagnosis of dementia compared to CRIS/OX (Fig. 4b)

### 3.3 Consistency of medication records between UKB and CRIS/OX

Of all 854 individuals, 129 had at least one agreed record of medications for mental health, and 272 individuals had no medication records on mental health disorders on either UKB or CRIS/OX, resulting in a matching rate of 47.0% (95% CI: 43.6–50.4%).

Of all patients that had matched diagnosis of the four main mental disorders of interest, the agreement on any antipsychotics was highest for schizophrenia (90.0%, 95% CI: 55.5–99.8%; Table 3), followed by bipolar disorder (66.7%, 95% CI: 46–83.5). Conversely, the agreement was very low for dementia (14.9%, 95% CI: 6.2–28.3%) which partially explained the overall low matching rate when any mental disorder was studied.

**Table 3:** Validation Results of Medications for Typical Mental Health Disorder Diagnosis.

	Dementia	Depression	Bipolar Disorder	Schizophrenia	Total
No. individuals in UKB	79	279	45	19	434
No. individuals in CRIS/OX	159	135	52	23	468
No. individuals with agreed diagnosis	47	35	27	10	149
No. individuals in agreed diagnosis with matched medications	7	17	18	9	61
Degree of agreement (PPV)	14.9%	48.6%	66.7%	90.0%	40.1%
95% CI	6.2–28.3%	31.4–66.0%	46.0–83.5%	55.5–99.8%	33.0–49.3%

We also compared the prevalence of individual drug prescription by disease diagnosis in the UKB vs. CRIS/OX (Table 4). For both UKB and CRIS/OX, there was relatively even split between frequently prescribed drugs with no specific medication dominating for any of the four listed mental health disorders. However, only a small number of medications matched between two data sources, including Donepezil for dementia, Venlafaxine for depression, Lithium and Quetiapine for bipolar disorder, Quetiapine and Olanzapine for schizophrenia.

**Table 4:** Most Frequently Prescribed Medications for Dementia, Depression, Bipolar Disorder, Schizophrenia in UKB and CRIS/OX.

	UKB				CRIS/OX	
	Drug Name	n	%	Drug Name	n	%
Dementia	Citalopram	4	16.00%	Mirtazapine	76	11.10%
	Amitriptyline	4	16.00%	Donepezil	57	8.30%
	Trihexyphenidyl	3	12.00%	Risperidone	47	6.90%
	Donepezil	2	8.00%	Zopiclone	45	6.60%
	Mirtazapine	2	8.00%	Olanzapine	43	6.30%

Depression	Citalopram	46	16.40%	Mirtazapine	78	11.50%
	Venlafaxine	33	11.80%	Venlafaxine	67	9.90%
	Fluoxetine	27	9.60%	Lithium	60	8.80%
	Amitriptyline	21	7.50%	Zopiclone	59	8.70%
	Lithium	17	6.10%	Olanzapine	49	7.20%

Bipolar Disorder	Lithium	18	20.90%	Lithium	42	11.50%
	Quetiapine	7	8.10%	Zopiclone	33	9.00%
	Sertraline	6	7.00%	Quetiapine	27	7.40%
	Venlafaxine	5	5.80%	Olanzapine	27	7.40%
	Lamotrigine	5	5.80%	Sodium Valproate	23	6.30%

Schizophrenia	Quetiapine	4	12.10%	Aripiprazole	14	7.90%
	Lithium	4	12.10%	Quetiapine	12	6.70%
	Citalopram	3	9.10%	Olanzapine	12	6.70%
	Procyclidine	3	9.10%	Risperidone	11	6.20%
	Olanzapine	2	6.10%	Clozapine	10	5.60%

### 3.4 Comparison of cognitive function tests

Both UKB and CRIS/OX have measures of cognitive function, indeed the EHR of CRIS/OX contain a battery of clinical tests used in individual assessments. However, we were not able to make a complete comparison due to time between test events. However, it is of note that the cognitive score from the HoNOS test was correlated to the four UKB cognitive function assessments extracted, Pairs Matching, Fluid Intelligence, Numeric Memory and Symbol Digit Substitution (Appendix E).

### 3.5 The distribution of individuals with the UKB linkage across the UK-CRIS network

In order to understand a more detailed distribution of individuals who have records in both UKB and CRIS, we approached a number of the CRIS network members and obtained their full agreement to share the number of individuals who have records in both CRIS and UKB. The resulting figures including the Oxford instance of CRIS are summarised in Table 5. This demonstrates the potential of multi-site linkage and enables power calculations for future studies.

**Table 5:** CRIS Network Member Trusts and the number of individuals with the linkage to UK Biobank.

CRIS Network Member	Individuals with UKB linkage
Oxford Health NHS Foundation Trust	854
South West London and St George's NHS Foundation Trust	1,053
Devon Partnership NHS Trust	72
Cumbria, Northumberland, Tyne & Wear NHS Foundation Trust	3,161
West London Mental Health Trust	1,450
Nottinghamshire Healthcare NHS Foundation Trust	3,964

## 4 Discussion

In this pilot linkage study of 854 individuals, we applied NLP methods to extract clinical information as a reference standard [8]. We validated three main categories of information commonly used for psychiatric research: demographic data, diagnosis outcomes and medication prescriptions in the UKB with a reference standard – CRIS/OX. We also explored the correlation amongst a range of cognitive tests between UKB and CRIS/OX (Appendix E). We found that: (1) the demographic information collected in the UKB had almost complete match with the reference standard; (2) the self-reported diagnostic data alone in the UKB identified less than half of the cases and individuals with the apparent diagnosis when other sources were included; (3) together with the HES inpatient psychiatric diagnoses, death registry and algorithmic-defined outcomes, the UKB data can be used with limited reliability to identify patients with mental health disorders. However, the degree of reliability varied by individual mental health disorders; (4) the self-reported medications from the UKB varied widely as compared to medications prescribed by clinicians; (5) there was no evidence that the cognitive tests presented in the UKB (Appendix E, F) appropriately reflect the performance of patients as measured by the clinically administered cognitive tests (such as MoCA and MMSE) presented in CRIS/OX.

We found that demographic information recorded in the UKB correlated strongly with that recorded in the CRIS/OX, confirming successful record linkage and suggesting that these data are likely to be accurate in the UKB. However, it is worth noting that there was a considerable amount of missing data on ethnic groups in CRIS/OX, limiting our ability to assess the validity of ethnicity related information in UKB. The lack of ethnicity information in CRIS/OX is primarily because such information was not requested by clinicians or not provided or even refused by patients. Data missingness is a common problem in the use of electronic health record [16] and comparison of linked data will aid researchers in interpretation and choices of imputation approaches.

It is interesting to note the differences in pseudo-anonymisation between the resources. Researchers implementing linkage between data sources should consider treating the anonymised data ethically and responsibly when using newly derived and augmented participant information. UKB has a specialised data sharing policy [17] whereas CRIS uses more stringent protection for its detailed, yet anonymised clinical records and great care should be taken to consider the most stringent guidelines at all times.

In terms of examination on diagnostic data, it is not surprising that UKB collected more subjects with diagnostic data of depression than CRIS/OX did, as most cases of depression may be treated at the primary care level and therefore not warrant referral to secondary care. For other types of typical mental health disorders, the discrepancies between data collected in the UKB and CRIS/OX are caused perhaps by the following two reasons: Firstly, when the baseline data collection was conducted between 2006 and 2010, participants might refuse or were reluctant to report their mental health issues due to worries of social discrimination or prejudice [18], [19]. For instance, only 3 (0.4%) cases of dementia from the cohort have been reported to UKB, which is considerably lower than the expected 1.3% prevalence in the general population reported in the UK [20]. Secondly, by using UKB hospital inpatient data, diseases that only required outpatient consultation or management in primary care are not sufficiently included. Moreover, the quality of HES data remains questionable including mental health reporting [21], [22]. Recently, primary care data were incorporated for a subset (∼45%) of the UKB population which will be a valuable resource for case ascertainment at the GP level [5].

At the time of study, only a small proportion of the participants recruited by UKB attended the follow-up assessment and updated their regular medication records. Reassuringly, UKB primary care records now contain prescribed medication information for a subset of the population [23]. Moreover, participants of UKB were specifically asked about mental health disorders in the Mental Health Questionnaire (MHQ) in August 2017. The outcomes of 31% of the UKB samples (157,366 responses) are now available [24]. However, there have been issues with the accurate interpretation of these new resources [24] and therefore the involvement of secondary resources such as CRIS with clinically created diagnosis for both validation and replication is of clear benefit. There is no doubt, once available for all individuals, these updates can serve as important complementary data to the current repository, assisting investigators in designing and performing advanced studies in mental health disorders.

This study has several limitations. Firstly, despite the decent performance of the NLP model for information extraction in precision and recall test [13], there may still exist discrepancies between the data retrieved from the NLP model, leading to biased results. Secondly, the time window for information collection by UKB and CRIS/OX were not perfectly coincided. Additionally, the proportion of participants recruited by UKB with follow-up information is relatively low (∼20%), although more assessments are to be included in further data releases. Finally, with the exception of the medication comparison, we limited our analysis to demographic and diagnosis with an aim at this stage to simply understand the validity of linkage and how it can be utilised in a wider clinical research question.

This study demonstrates multiple avenues for further investigation. Recently, CRIS and UKB have established a linkage of a total number of 15,000 participants across CRIS network members, providing a unique opportunity for conducting studies using all linked subjects. The detailed information about the distribution of individuals within each of the CRIS network member is presented in Table 5. The next step will involve employing the developed analytical approach, including NLP models, to extract more detailed phenotypic information to allow characterisation of individuals and advanced analysis with greater depth. However the nature of the electronic health records also brings particular challenges which should be addressed adequately in order to maximise their secondary use for research, including the proper treatment of missing records, mitigating selection biases of various data fields, data harmonisation and normalisation of variable names across different electronic patient record systems, such as CareNotes and Rio [25]. Furthermore, the longitudinal nature of heterogeneous variables extracted from secondary electronic health records allows the development of a high-dimensional patient trajectory which might be effectively represented by means of the signature transformation [26] and used for various downstream tasks with recurrent neural networks [27] or Transformer-based architectures [28].

Both data sources, UKB and CRIS, represent untapped potential for comprehensive research in mental health, synergistically complementing each other in various data modalities. Specifically, it is now possible to combine precise information from UKB, such as data from wearable devices, imaging and genetic data with accurate and clinically validated longitudinal data from CRIS, including diagnoses, treatments, administered medications, psychological sessions and information from specialised clinics.

In the first study of linkage between two major UK data resources in mental health, our validation results suggested that by combining data records we can give a more comprehensive patient view both over time and with respect to phenotypic characteristics. Further understanding between differences recorded and integration of primary care will strengthen these resources.

## Declaration of Competing Interest

The authors declare that they have no known competing financial interests or personal relationships that could have appeared to influence the work reported in this paper.
