# Supplementary material for: Validation of UK Biobank data for mental health outcomes: A pilot study using secondary care electronic health records
Source: Int J Med Inform. 2022 Apr;160:104704. doi: 10.1016/j.ijmedinf.2022.104704 (PMC8889024; doi:10.1016/j.ijmedinf.2022.104704)
Supplement: Supplementary data 1 [file mmc1.docx]

**Appendix A: Procedure of Record Linkage**

The procedure of record linkage between UKB and CRIS/OX involves following steps:

1. Apply for UKB data with linkage permissions.
2. Apply for research passport as a CRIS researcher.
3. Apply for registration of research projects using data from CRIS/OX.
4. Retrieve a list of individuals in CRIS/OX with linkage to UKB.
5. Submit to UKB with identification hash codes provided by CRIS/OX.
6. Select participants from UKB with matched hash codes.

**Appendix B: Diagnosis Outcome in UKB**

Diagnostic disease outcomes in the UKB are acquired from multiple sources:

1. **Self-reported disease:** Participants informed any medical conditions at the baseline UKB interview. Self-reported diseases were originally encoded in 4-digit format according to a coding list pre-defined by UKB group and then transformed into corresponding International Classification of Disease 10^th^ Revision (ICD-10) codes prior to performing the matching procedure.
2. **Hospital Episode Statistics (HES) Inpatient Data:** UKB has obtained routinely-collected coded clinical healthcare datasets [1] via linkages to national hospital admissions for all participants. Cases were determined as having ever received an ICD-9 and ICD-10 code relating to any mental health issues in main or secondary diagnosis. All the ICD-9 codes are transformed to corresponding ICD-10 codes based on opinions from clinical professionals.
3. **Mortality data:** Primary and secondary causes of death reported for participants are collected by linkages to mortality registries and recorded in the format of ICD-10 codes.
4. **Algorithmic-defined outcomes (only for dementia):** Besides the above mentioned sources of diagnostic data, UKB has also independently defined a rule based algorithm to identify participants with different types of dementia. Data sources on which the algorithm relies are UKB baseline assessment data, HES data and death register data.

**Appendix C: Dictionary for Transformation of 4-digit Self-report Disease Code to ICD-10 Code**

| **Disease Description** | **4-Digit Code in UKB** | **Group of ICD-10 Codes** |
| --- | --- | --- |
| Dementia | 1263 | F00, F01, F02, F03, G30 |
| Depression | 1286 | F32, F33 |
| Post-natal Depression | 1531 | F530 |
| Bipolar Disorder | 1291 | F31 |
| Schizophrenia | 1289 | F20, F21, F231, F232, F25, F601 |

Table C.1: Dictionary for Transformation of 4-digit Self-report Disease Code to ICD-10 Code

When an individual is recorded with one of the 4-digit codes listed in Table C.1, the code is transformed to the corresponding group of ICD-10 codes for comparison.

Table C.2 depicts the distinct levels of precision for diagnosis with their corresponding presentation of ICD-10 codes [2].

| **Level** | **Description** | **Explanation** | **Example** |
| --- | --- | --- | --- |
| Top level  (LEVEL-1) | Blocks of 3-character categories | General diagnosis | F00 - F09 Organic, symptomatic mental health disorders |
| Medium level  (LEVEL-2) | 3-character categories  (1 alphabet with 2 digits) | Specific diagnosis | F01  Vascular dementia |
| Bottom level  (LEVEL-3) | 4-character subcategories  (1 alphabet with 3 digits) | Detailed diagnosis | F001  Vascular dementia of acute onset |

Table C.2: ICD-10 Codes with Different Levels

**Appendix D: Comparison of Gender Information between UKB and CRIS/OX**

Figure D.1 depicts the contingency table showing complete matching for gender demographic


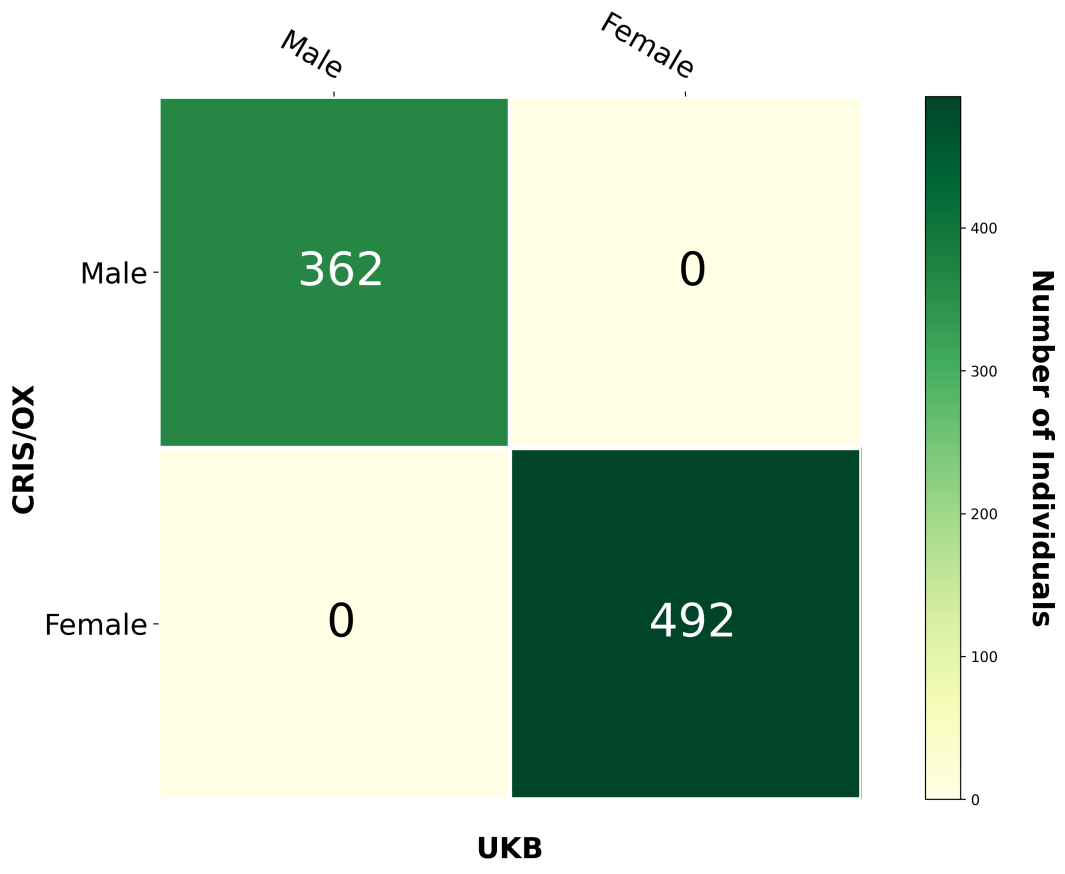


Figure D.1: Contingency Table for Gender Demographic

**Appendix E: Comparison of Cognitive Assessment Tests**

For measure of the relationship between each of the pairwise tests (a pair of cognitive tests in UKB and CRIS/OX), the correlation is measured by the simple Pearson Correlation Coefficient along with the corresponding *p*-value. Essentially, UKB implements a battery of cognitive function tests as part of the participant’s baseline visit to the assessment centre and follows up with online testing [3]. For the cohort under study, the most frequent cognitive tests across participants are pairs matching test, fluid intelligence test, symbol digit substitution test and numeric memory test (consult Appendix F for more details) [4]. From CRIS/OX, records of clinical assessments with used for diagnosis of mental health problems, including cognitive tests in HoNOS, MoCA and MMSE, are extracted from collected clinical notes with related attachments. Note that only the results of follow-up cognitive tests in the UKB were employed for correlation calculation, as the average time gap between baseline assessment for UKB and medical tests recorded in CRIS/OX is relatively huge, leading bias to the computation results. Additionally, each individual might take part in the same medical test several times during their multiple visits to medical care services. The statistical indices reported were based on the results of the medical test on the nearest date to their regular cognitive tests. All the listed medical tests were performed within an average of +/- 2.5 years from the medical tests.

Although the number of individuals who attend both the medical tests and simple cognitive tests is relatively small, Table E.1 depicts that both MoCA and MMSE scores has no significant correlation to the outcomes of regular cognitive tests, whereas the cognitive test result in HoNOS shows decent linear relationship to its counterparts for regular simple tests, which is consistent with human common sense.

It is natural to believe that the simple cognitive test proposed in the UKB cannot be comparable with the clinical tests such as MoCA and MMSE, since each of these cognitive tests only enables to disclose one of the functionalities for individuals, without capability of revealing the comprehensive cognitive status. An advanced investigation including rigorous selection of sections from medical tests and comparison to the relevant simple cognitive tests is worth attempting for further study.

|  | | **Pairs**  **Matching** | **Fluid Intelligence** | **Numeric Memory** | **Symbol Digit Substitution** |
| --- | --- | --- | --- | --- | --- |
| **HoNOS  (Cognitive)** | *r* | 0.4637 | -0.4741 | -0.5126 | -0.3738 |
|  | *p*-value | 0.0057 | 0.0026 | 0.0032 | 0.027 |
|  | Time gap (years) | 2.4 | 2.4 | 2.4 | 2.4 |
|  | No. individuals (n) | 34 | 38 | 31 | 35 |
| **MoCA** | *r* | -0.1446 | 0.2574 | 0.3276 | -0.0180 |
|  | *p*-value | 0.3732 | 0.1136 | 0.0586 | 0.9160 |
|  | Time gap (years) | 2.2 | 2.2 | 2.2 | 2.2 |
|  | No. individuals (n) | 40 | 39 | 34 | 37 |
| **MMSE** | *r* | -0.3701 | 0.1278 | 0.3135 | 0.2001 |
|  | *p*-value | 0.0686 | 0.5519 | 0.1554 | 0.3846 |
|  | Time gap (years) | 2.2 | 2.2 | 2.2 | 2.3 |
|  | No. individuals (n) | 25 | 24 | 22 | 21 |

Table F.1: Correlation Test Results for Medical Test and Regular Cognitive Test

**Appendix F: Description of cognitive tests in UKB**

**Pairs matching test:** a participant is asked to memorise the position of as many matching pairs of cards as possible. The cards are then turned face down on the screen and the participant is asked to touch as many pairs as possible in the fewest attempts. The number of incorrect attempts is recorded.

**Fluid intelligence test:** a participant has 2 minutes to complete as many questions as possible from the test. The number of correct answers to the questions is recorded.

**Numeric memory test:** a participant was shown a 2-digit number to remember initially. The number then disappeares and after a short while he/she is asked to enter the number onto the screen. The number became one digit longer each time he/she remembered correctly (up to a maximum of 12 digits). The maximum number of digits remembered is recorded.

**Symbol digit substitution test:** a participant is presented with a series of grids in which symbols are to be matched to numbers according to a key presented on the screen. The number of symbol digit matches made correctly is recorded.

**Reference**

1. Benchimol EI, Smeeth L, Guttmann A, Harron K, Moher D, Petersen I, Sørensen HT, von Elm E, Langan SM, Committee RW: **The Reporting of studies Conducted using Observational Routinely-collected health Data (RECORD) statement**. *PLoS medicine* 2015, **12**(10):e1001885.

2. WHO: **International statistical classiﬁcation of diseases and related health problems**. In*.*, vol. 2, Fifth edn; 2016: 17.

3. Calvin CM, Wilkinson T, Starr JM, Sudlow C, Hagenaars SP, Harris SE, Schnier C, Davies G, Fawns-Ritchie C, Gale CR *et al*: **Predicting incident dementia 3-8 years after brief cognitive tests in the UK Biobank prospective study of 500,000 people**. *Alzheimers Dement* 2019, **15**(12):1546-1557.

4. **Cognitive function online** [<http://biobank.ndph.ox.ac.uk/showcase/label.cgi?id=116>]
